# Adaptive Control of the Meiotic Recombination Landscape by DNA Site-dependent Hotspots With Implications for Evolution

**DOI:** 10.3389/fgene.2022.947572

**Published:** 2022-06-22

**Authors:** Reine U. Protacio, Mari K. Davidson, Wayne P. Wahls

**Affiliations:** Department of Biochemistry and Molecular Biology, University of Arkansas for Medical Sciences, Little Rock, AR, United States

**Keywords:** meiosis, recombination evolution, recombination hotspot, linkage disequiblibrium, genetic mapping, evolution, Schizosaccharomyces pombe

## Abstract

Meiosis is an essential component of the sexual life cycle in eukaryotes. The independent assortment of chromosomes in meiosis increases genetic diversity at the level of whole chromosomes and meiotic recombination increases genetic diversity within chromosomes. The resulting variability fuels evolution. Interestingly, global mapping of recombination in diverse taxa revealed dramatic changes in its frequency distribution between closely related species, subspecies, and even isolated populations of the same species. New insight into mechanisms for these evolutionarily rapid changes has come from analyses of environmentally induced plasticity of recombination in fission yeast. Many different DNA sites, and where identified their binding/activator proteins, control the positioning of recombination at hotspots. Each different class of hotspots functions as an independently controlled rheostat that modulates rates of recombination over a broad dynamic range in response to changing conditions. Together, this independent modulation can rapidly and dramatically alter the global frequency distribution of recombination. This process likely contributes substantially to (i.e., can largely explain) evolutionarily rapid, Prdm9-independent changes in the recombination landscape. Moreover, the precise control mechanisms allow cells to dynamically favor or disfavor newly arising combinations of linked alleles in response to changing extracellular and intracellular conditions, which has striking implications for the impacts of meiotic recombination on evolution.

## Introduction

With few exceptions, eukaryotes have an obligate sexual life cycle with alternating haploid and diploid states; these are coupled by meiosis and fertilization. Homologous recombination is induced to high levels in meiosis and serves two key functions (Henderson and Bomblies, 2021). First, meiotic recombination is generally required for the proper segregation of chromosomes in meiosis and, correspondingly, for the faithful transmission of chromosomes between generations. Second, recombination shuffles the linkages of gene-alleles within chromosomes. The independent assortment of chromosomes in meiosis promotes genetic diversity at the level of whole chromosomes and meiotic recombination promotes genetic diversity within chromosomes; together, these processes provide genetic variability that is the fuel for evolution. Notably, meiotic recombination is the only way to uncouple newly arising, *cis*-linked, deleterious mutations and to generate new combinations of beneficial alleles within the same chromosome (Otto, 2021). To adapt a quote from Lewis Thomas (Thomas, 1979), if there were no meiotic recombination, we would still be anaerobic bacteria and there would be no music.

### Hotspots Regulate Frequency Distribution of Recombination

**FIGURE 1:**
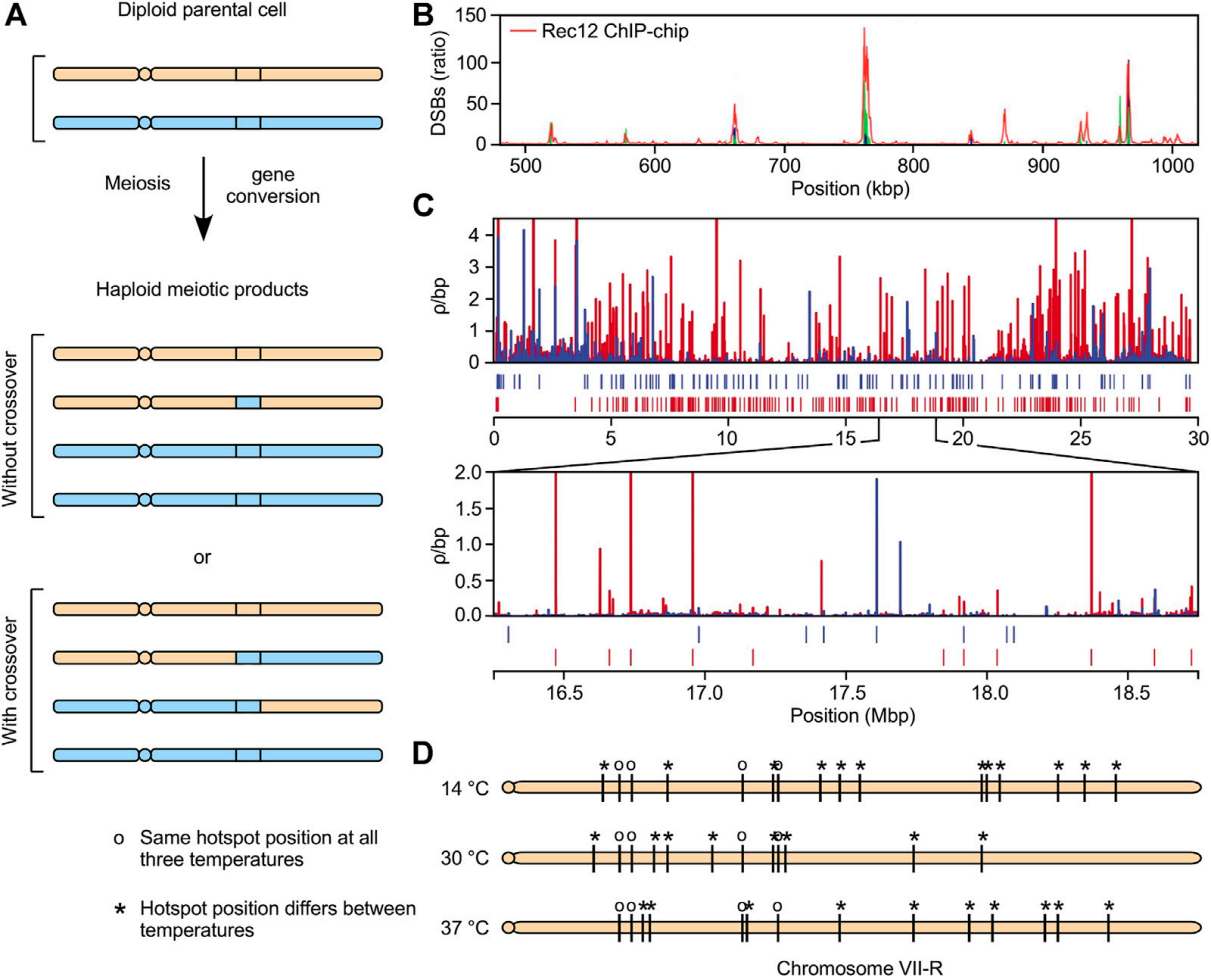
Highly dynamic, extensive changes in the meiotic recombination landscape **(A)** Meiotic recombination leads to gene conversion, with or without reciprocal exchanges (crossovers) **(B)** Meiotically induced, recombination-initiating dsDNA breaks (DSBs) cluster at hotspots that position recombination in the genome. This example depicts frequency distribution of DSBs along a portion of chromosome one in fission yeast **(C)** Example of evolutionarily rapid changes. Plot shows distribution of recombination rates along chromosome one in two different populations (*red, blue*) of stickleback fish, along with positions of identified hotspots (*tic marks*) **(D)** Example of environmentally induced changes. Plot shows effects of temperature on DSB hotspot positions (*tic marks*) on right arm of chromosome seven in budding yeast. We posit that the dramatic, evolutionarily rapid (panel C) and environmentally induced (panel D) changes in the recombination landscape share a common molecular mechanism.

Meiotic recombination is controlled at multiple levels including where it initiates, how pathway intermediates are resolved, and the modulation of average rates along entire chromosomes in response to chromosome size (Zelkowski et al., 2019; Sanchez et al., 2021; Yadav and Bouuaert, 2021). As with many biochemical pathways, a crucial, rate-limiting step occurs early in the pathway; specifically, the induction of recombination-initiating dsDNA breaks (DSBs). These DSBs are created by the meiotically induced, basal recombination machinery *via* its catalytic subunit, Spo11/Rec12 (Lam and Keeney, 2014; Robert et al., 2016; Jing et al., 2019). The broken chromosome is repaired using its homolog (or sister chromatid) as a template. When the repair events encompass heterologies (such as single-nucleotide polymorphisms, or SNPs), they can produce scorable recombination events. These manifest primarily as gene conversion events, a subset of which are accompanied by reciprocal recombination events (crossovers) between the homologs (Figure 1A) (Lorenz and Mpaulo, 2022). Correspondingly, there is good agreement between mapped distributions of DSBs, gene conversions, and conversion-associated crossovers across the genome.

Genetic approaches to map global frequency distributions of recombination each involve the genotyping (by deep sequencing or DNA microarray hybridization) of many genomes to detect, and measure or infer with precision, rates of recombination between markers (e.g., SNPs) along chromosomes (Penalba and Wolf, 2020). There are also molecular approaches to map the distribution of recombination-initiating DSBs (e.g., Figure 1B) (Gerton et al., 2000; Blitzblau et al., 2007; Pan et al., 2011). These DSB-mapping approaches, so far applied to a limited but growing number of species, have higher resolution and sensitivity than the genetics-based approaches, whose resolution is limited by the density and distribution of genetic markers, such as SNPs. Regardless of approach, mapping of recombination in diverse taxa has revealed that recombination is clustered preferentially at discrete locations, called hotspots, which control the frequency distribution of recombination across the genome (Wahls and Davidson, 2012; Choi and Henderson, 2015; Ergoren, 2018).

It is standard practice in the field to annotate the positions of hotspots based on some type of frequency cutoff threshold (i.e., a given location in the genome is judged to be either “hot” or “not hot”). However, there are differences in the sensitivities and resolutions of the different mapping approaches that are used. Moreover, hotspot calling criteria differ substantially among studies. Such factors can complicate the interpretation of hotspot maps. More fundamentally, the process of annotating the positions of hotspots can be misleading because—as described subsequently in this perspective—individual hotspots actually function as rheostats that can variably modulate rates of recombination over a broad dynamic range (Brown et al., 2020; Protacio et al., 2022). From our perspective, these factors are germane to long-recognized, mechanistically enigmatic features of the meiotic recombination landscape.

### Enigma 1: Evolutionarily Rapid Redistribution of Recombination

One might expect that the global frequency distribution of meiotic recombination would differ substantially between highly diverged taxa. Conversely, one might expect recombination landscapes to be similar between closely related species. Intriguingly, and contrary to expectations, analyses conducted in diverse taxa have revealed striking differences in fine-scale recombination maps and annotated hotspot positions between closely related species, sub-species and isolated populations of the same species.

An example of this phenomenon is provided in Figure 1C. This plot shows the frequency of crossover recombination per unit distance, calculated using linkage disequilibrium (LD) values, in two different populations of stickleback fish that live in distinct environments (marine and freshwater) (Shanfelter et al., 2019). There are profound differences in the recombination landscapes between these two populations. Moreover, as is standard practice in the field, the authors used a peak calling algorithm to annotate the positions of recombination hotspots. This approach revealed that about 85% of identified hotspots are at different positions in the two populations—even though those populations only became isolated from each other within the past 15 thousand years.

Similar differences have been documented in other taxa. For example, in flycatcher birds 55%–61% of annotated hotspots are at different positions between pairs of closely related species, and 31%–49% of hotspot positions differ between isolated populations of the same species (Kawakami et al., 2017). Likewise, about 80% of inferred hotspots are at different locations in sub-species of rice (Marand et al., 2019). Similarly, the distribution of annotated hotspots differs substantially between species of the yeast genus *Lachancea*; as well as between *Lachancea sp* and *Saccharomyces sp* (Brion et al., 2017). There are also differences in hotspot positions between species of the genus *Saccharomyces* (Tsai et al., 2010; Lam and Keeney, 2015). Mechanisms for such evolutionarily rapid, Prdm9-independent changes in the global frequency distribution of meiotic recombination across different kingdoms of the eukaryotic domain were unknown. From our perspective, the mechanisms are related to those of another interesting phenomenon; namely, environmentally induced plasticity of recombination.

### Enigma 2: Environmentally Induced Redistribution of Recombination

More than a century ago, it was discovered that extrinsic factors (e.g., temperature) and intrinsic factors (e.g., genetic differences) affect rates of meiotic recombination (Plough, 1917; Sturtevant, 1917). Subsequent studies revealed that such plasticity is common, if not ubiquitous, across taxa. Intrinsic factors such as genetic background, sex, mating type, auxotrophies, and DNA sequence polymorphisms can each affect rates and patterns of recombination (Szankasi et al., 1988; Parvanov et al., 2008; Kong et al., 2010; Hyppa et al., 2014; Brick et al., 2018). Extrinsic factors such as temperature, nutrients, osmolarity, stress and parasite infection can also affect recombination (Abdullah and Borts, 2001; Parvanov et al., 2008; Cotton et al., 2009; Stahl et al., 2016; Singh, 2019; Brown et al., 2020). Most of the work on the effects of environmental conditions (e.g., temperature) on recombination has been conducted at low resolution (e.g., by counting chiasmata, which are cytological manifestations of crossovers) or by analyzing frequencies of DSBs and/or rates of recombination at a limited number of locations in the genome. However, in a few cases, the impacts of environmental conditions on the global frequency distribution of recombination has been determined at high resolution under well-controlled laboratory conditions.

A striking example of genome-wide plasticity came from determining the effects of temperature on the distribution of DSB hotspots, as defined using a frequency cutoff threshold, in budding yeast (Figure 1D) (Zhang et al., 2017). Remarkably, only about 20% of annotated hotspots throughout the genome were at the same positions at all three temperatures; the positions of ∼80% of hotspots differed between two or all three temperatures. The conclusions are unambiguous: changing environmental conditions can trigger dramatic, genome-wide changes in the fine-scale recombination landscape.

As is the case for the evolutionarily rapid changes in the recombination landscape described above, mechanisms for environmentally induced plasticity of recombination have been elusive. One long-standing notion is that the synaptonemal complex (SC) might contribute to such plasticity [see reviews by (Morgan et al., 2017; Henderson and Bomblies, 2021)]. However, while changes in the SC could affect recombination rates at broad scale (e.g., at the level of whole chromosomes), it is difficult to envision how the SC could mediate dramatic changes in the fine-scale recombination landscape (e.g., Figures 1C,D). Moreover, organisms that lack an SC, such as fission yeast (Kohli and Bahler, 1994), still have fine-scale plasticity (Hyppa et al., 2014; Brown et al., 2020; Protacio et al., 2022). Such localized changes in recombination rates—which are increased at some sites and decreased at others—would require perforce a means to dynamically and differentially control rates of recombination locally; i.e., at the resolution of individual recombination hotspots.

### Discrete DNA Sites Position Recombination at Hotspots

**FIGURE 2:**
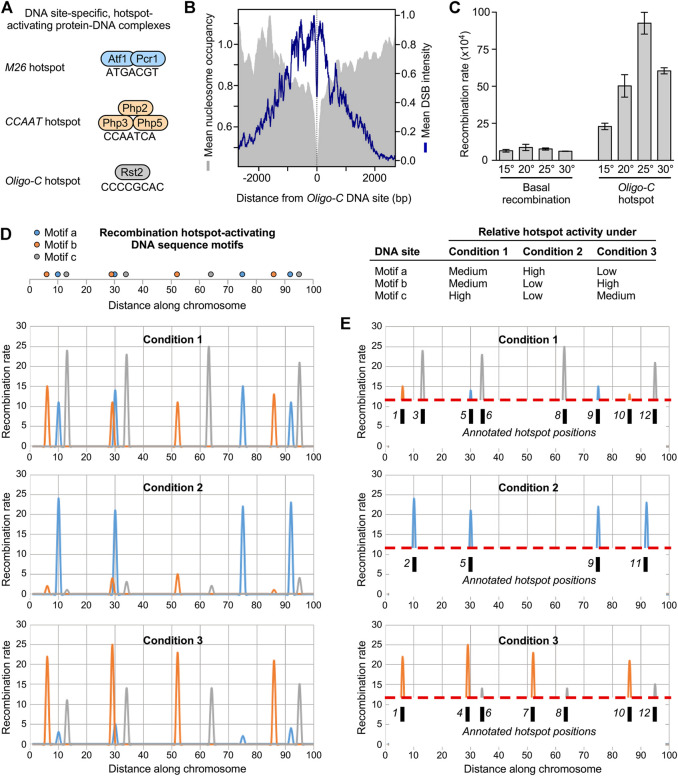
Mechanisms for plasticity in the frequency distribution of meiotic recombination **(A)** Examples of DNA site-specific protein-DNA complexes (*cis-*acting regulatory modules) that activate recombination hotspots **(B)** The *cis-*acting regulatory modules promote catalysis of DSBs by the basal recombination machinery. In this example, *Oligo-C* DNA sites in the genome were identified, oriented and aligned; plot shows average distribution of DSBs (*blue*) and nucleosomes (*grey*) around the DNA site **(C)** Hotspots function as rheostats that variably modulate recombination rates in response to extracellular and intracellular cues. Example shows rates of recombination for the *Oligo-C* hotspot and for a basal recombination control at different temperatures **(D)** Model for plasticity in the recombination landscape. Each hotspot DNA site promotes recombination in its vicinity. Each different class of hotspots is independently controlled in response to changing conditions. Their net contributions under different conditions dramatically alter the recombination landscape **(E)** It is standard practice in the field to apply frequency thresholds (illustrated by *dashed red lines*) to annotate hotspot positions (*numbered tic marks*). This can give the false impression that the positions of hotspots “move” from condition to condition or population to population (compare E to D).

To identify mechanisms that control the redistribution (plasticity) of meiotic recombination, one must first identify what controls its positioning in the genome. In other words, what makes a hotspot hot? The primary, *cis*-acting determinants are unknown in most species. However, by focusing on an allele-specific hotspot in fission yeast (Gutz, 1971), Jürg Kohli’s lab discovered the DNA site-dependent control of hotspots (Schuchert et al., 1991). Contemporaneously, Tom Petes’ group provided evidence for such regulation in budding yeast (White et al., 1991; White et al., 1993). Subsequently, using an insightful genetic screen in fission yeast, Walter Steiner’s lab identified about 200 additional, distinct, short (30 base pairs or less), hotspot-activating DNA sequences (Steiner et al., 2009). Five of the regulatory DNA sequence motifs (DNA sites) have been defined with precision by systematic, comprehensive, base pair substitutions in the genome (Schuchert et al., 1991; Steiner et al., 2009; Steiner et al., 2011); and the binding/activator protein complexes have been identified for three of these DNA sites (Figure 2A) (Wahls and Smith, 1994; Kon et al., 1997; Steiner et al., 2011). The different classes of DNA site-dependent hotspots each function by a common downstream effector mechanism. They each trigger the displacement of nucleosomes to increase access for the basal recombination machinery to its DNA substrates in chromatin (Storey et al., 2018; Mukiza et al., 2019), thereby stimulating the frequency of recombination-initiating DSBs (e.g., Figure 2B) (Steiner et al., 2002; Wahls and Davidson, 2010; Fowler et al., 2014).

Hotspot-activating DNA sites and binding/activator proteins discovered in fission yeast are conserved functionally in its distant cousin, budding yeast (Steiner and Steiner, 2012; Wahls and Davidson, 2012). This is notable because fission yeast and budding yeast are about as evolutionarily distant from each other as humans are from nematodes (Sipiczki, 2000; Heckman et al., 2001). Moreover, hotspot-associated DNA sites identified computationally from recombination maps in other taxa, such as honeybees (Mougel et al., 2014), are identical or similar to hotspot-activating DNA sites of fission yeast. Thus, DNA site-dependent mechanisms for the positioning of meiotic recombination are likely broadly conserved. In support of this idea, downstream effector mechanisms discovered in fission yeast [e.g. (Mizuno et al., 1997; Yamada et al., 2004; Hirota et al., 2007),] are also conserved. For example, diverse species exhibit the displacement of nucleosomes at or near hotspot centers [reviewed by (Tock and Henderson, 2018), which is a fundamental characteristic of, and known effector mechanism for, DNA site-dependent hotspots [see Figure 2B and (Mizuno et al., 1997; Mukiza et al., 2019)]. As a more discrete example of conserved effector mechanisms, orthologs of an ATP-dependent chromatin remodeling enzyme of fission yeast (Snf22) and mice (Hells) are each recruited to, and help to activate *via* nucleosome displacement, DNA site-dependent hotspots (Yamada et al., 2004; Storey et al., 2018; Mukiza et al., 2019; Spruce et al., 2020).

Nearly two decades after the DNA-site dependent activation of hotspots was discovered in fission yeast (Schuchert et al., 1991), it was implicated by association in mammals (Myers et al., 2008). In a subset of metazoans (e.g., mice, cattle and humans), Prdm9 binding sites can activate hotspots [see reviews by (Grey et al., 2018; Paigen and Petkov, 2018)]. Because this mechanism operates in humans and the DNA binding domain of Prdm9 evolves rapidly, it has garnered much attention. However, a far greater diversity of species (e.g., fungi, birds, amphibians, many fishes, canids, marsupials and plants) lack Prdm9 or one of its functionally important domains, but still have hotspots that tend to distribute in a more yeast-like fashion in the genome [e.g. (Munoz-Fuentes et al., 2011; Axelsson et al., 2012; Kawakami et al., 2017),]. Interestingly, Prdm9-expressing species can still have hotspots that are located remote from Prdm9 binding sites (Schield et al., 2020). Moreover, upon the ablation of Prdm9 in mice and rats hotspots are not eliminated; instead, they adopt a more yeast-like distribution in the genome (Brick et al., 2012; Mihola et al., 2021). Such findings suggest that there is an evolutionarily ancient, Prdm9-independent mechanism for distributing recombination to hotspots. The functionally conserved, *cis*-acting regulatory elements discovered in fission yeast provide such a mechanism (Wahls and Davidson, 2012).

In summary, paradigms from fission yeast (e.g., about 200 different, short DNA sequences are known to directly activate hotspots) and data from other species each support the original, evidence-based hypothesis that much, if not most meiotic recombination is positioned in the genome by hotspot-activating DNA sites and their binding proteins (Wahls and Smith, 1994).

### Hotspot-Activating DNA Sites Directly Control Plasticity of Recombination

If most meiotic recombination is positioned in the genome by hotspot-activating DNA sites, then those DNA sites might directly control plasticity of the recombination landscape. A recent study took advantage of the powerful fission yeast model system to test this hypothesis (Protacio et al., 2022). That study determined the effects of changes in three different environmental conditions (temperature, carbon source and osmolarity) on rates of recombination at a test locus that contained one of three hotspot-activating DNA sites (*M26*, *CCAAT* or *Oligo-C*) or a basal recombination control that lacked those DNA sites. This approach revealed that the impacts of changing environmental conditions on local rates of recombination are mediated directly and primarily by DNA site-specific hotspots and their binding/activator proteins. Key findings include the following.

First, changing environmental conditions has little or no impact on rates of basal (hotspot-independent) recombination. Second, DNA site-dependent hotspots function as rheostats that variably modulate rates of recombination over a broad dynamic range in response to changing conditions (e.g., Figure 2C). A given hotspot can range from being quiescent (i.e., not promote recombination beyond basal levels) to being highly recombinogenic (i.e., greatly stimulate the initiation of recombination by the basal recombination machinery). Third, each different class of DNA site-dependent hotspots functions as an independently controlled rheostat. For example, under a given set of conditions one type of hotspot can promote recombination substantially while another type of hotspot is quiescent. As another example, a discrete change in the environment that increase the activity of one class of hotspots can decrease the activity of another class.

The new discoveries support a model for environmentally induced plasticity of the meiotic recombination landscape (Figure 2D) (Protacio et al., 2022). Each class of hotspot-activating DNA sites functions as an independently controlled rheostat that modulates rates of recombination at its own locations in the genome in response to its own constellation of signals. This independent modulation of rates by many different classes of DNA sites provides a molecular mechanism for precisely controlled, highly dynamic, large-scale changes in the global frequency distribution of meiotic recombination.

The findings also have important implications for hotspot annotation and interpretations therefrom. It is standard practice in the field to define the positions of hotspots by applying frequency cutoff thresholds to recombination maps. However, individual DNA site-specific hotspots actually control recombination rates over a broad dynamic range in response to changing conditions (Protacio et al., 2022). Consequently, the process of annotation can give the false impression that the positions of hotspots “move” from sample to sample, even when the positions of the hotspot regulating DNA sites have not changed (illustrated in Figure 2E). From our perspective, such factors force a shift in thinking about—and reveal fundamental mechanisms for—meiotic recombination-mediated dynamics of genomes, as well as the interplay between meiotic recombination and evolution.

### Implications for Genome Dynamics and Evolution

The substantial differences between recombination landscapes of closely related species, sub-species and isolated populations of species that lack Prdm9 have been baffling. Given the low amounts of overall DNA sequence divergence (e.g., between populations), it is implausible that the differences in recombination stem from large-scale changes in the sequences of hotspot-regulating DNA sites throughout the genome or in the DNA binding site specificities of the various binding/activator proteins. The discovery of mechanisms for environmentally induced plasticity (Protacio et al., 2022) provides solutions to this quandary.

First, many of the observed differences (e.g., between populations) might stem from environmentally induced, hotspot DNA site-dependent changes in the recombination landscape. For example, the differences in LD-based recombination maps between freshwater and marine populations of stickleback fish (e.g., Figure 1C) might be due largely to the differences in their environments (e.g., Figure 1D). A testable prediction of this hypothesis is that changing the environmental conditions would trigger substantial changes in distributions of recombination within each population; and that those changes would affect rates of recombination at most of the locations where substantial, population-dependent differences in the recombination landscape have been observed. Such a test would require a more direct approach to map recombination than the widely employed, LD-based approach, which infers average historical rates of recombination over many generations (Penalba and Wolf, 2020).

Second, the molecular mechanisms for plasticity also provide a way to fix (*via* genetic drift) some of the changes in recombination landscapes during isolation, divergence and speciation. Initial clues for this came from the *M26* class of DNA site-specific hotspots. As one might expect based on their response to changing environmental conditions (Brown et al., 2020; Protacio et al., 2022), *M26*-class hotspots are controlled by components of several different signal transduction pathways that respond to environmental and metabolic cues (Kon et al., 1998; Mizuno et al., 2001; Hirota et al., 2003; Hirota et al., 2004; Yamada et al., 2004; Hirota et al., 2007; Hirota et al., 2008; Gao et al., 2009; Storey et al., 2018). Extending such analyses to multiple different classes of DNA site-specific hotspots was particularly informative. As is the case for the different environmental cues, individual mutations in different components of the signaling pathways affect differentially rates of recombination among distinct classes of hotspots (Mukiza et al., 2019; Protacio et al., 2022). One point in particular—that as little as a single heterology in the genome (e.g., one mutation in a signal transduction pathway) can be sufficient to strongly and differentially adjust the distinct hotspot rheostats—has profound implications. Even minor genetic differences between species, subspecies and isolated populations can, by affecting signal transduction networks that differentially control distinct classes DNA site-specific hotspots, trigger substantial changes in the distribution of recombination across the genome (Protacio et al., 2022). Correspondingly, factors such as genetic drift, bottlenecks, loss and fixation of polymorphisms that affect signal transduction networks would each contribute to changes in the recombination landscape during isolation and speciation—mediated ultimately and directly through the hotspot-regulating DNA sites and their respective binding/activator proteins. Notably, such evolution in the recombination landscape would not require any changes in the distribution of the regulatory DNA sites or in their binding/activator proteins.

A third DNA site-dependent mechanism for changing the recombination landscape over evolutionary time scales involves the loss and gain of hotspot-activating DNA sites themselves (Wahls and Davidson, 2011). Because recombination hotspots serve preferentially as recipients of genetic information during gene conversion (Gutz, 1971), they are inherently suicidal. For example, DNA site-dependent hotspots promote the formation of recombination-initiating DSBs in their vicinity (e.g., Figure 2B) (Steiner et al., 2002; Wahls and Davidson, 2010; Fowler et al., 2014). When the hotspot DNA site is heterozygous, resection of the DSB and repair of that break from the homologous chromosome template will tend to remove the regulatory DNA site. The finding that all well-defined, hotspot-activating DNA sites are substantially under-represented in the genome provides evidence for such meiotic drive towards loss over evolutionary time scales (Wahls and Davidson, 2011). But despite their suicidal tendencies, hotspots have persisted in genomes (albeit not necessarily at their ancestral locations); this has been called the “hotspot paradox” (Boulton et al., 1997). Notably, the generation of hotspot DNA sites by mutations provides a solution to this paradox. For any given hotspot-activating DNA site, there is a large number of inactive, closely related, “cryptic” permutations of that DNA site which can be rendered active by as little as a single base pair substitution. Such cryptic DNA sites are abundant in the genome; e.g., using just five of the hotspot-activating motifs discovered by Steiner et al. (Steiner et al., 2009), we identified 64,622 cryptic motifs with average spacing of one every 194 base pairs (Wahls and Davidson, 2011). Similar considerations apply for the hundreds of other hotspot-activating DNA sequence elements (by extension, cryptic motifs would be ubiquitous and densely packed in the genome). Consequently, each spontaneous mutation has a remarkably high probability of generating a hotspot-activating DNA site. Thus, an equilibrium between rates of hotspot loss (*via* gene conversion) and hotspot gain (*via* mutations) would contribute to the retention and repositioning of DNA site-dependent hotspots over evolutionary time scales (Wahls and Davidson, 2011). Presumably, selective pressures that impinge upon the *cis*-acting regulatory modules also influence the retention, loss and gain of hotspots at various locations in the genome over time.

Lastly, meiotic recombination fuels evolution by uncoupling newly arising, *cis*-linked, deleterious mutations and by generating new combinations of beneficial alleles within the same chromosome (Otto, 2021). The ability to rapidly, precisely and extensively remodel the recombination landscape provides a way to favor or disfavor newly arising combinations of alleles in response to changing intracellular and extracellular conditions (Protacio et al., 2022). Hypothetically, this form of precisely controlled adaptation confers selective advantages to cells in meiosis, or at subsequent vegetative stages of the life cycle, or both. In support of this idea, meiotic recombination hotspots contribute to combinatorial diversity within the major histocompatibility complex (MHC) and, moreover, hotspot usage varies among populations (Fulton et al., 2016; Beeson et al., 2019; Sun et al., 2022). Thus, the presence of recombination hotspots within the MHC promotes, and can variably control, the diversity of haplotypes that confer self/non-self recognition and adaptive functions of the immune system. We posit that such changes in meiotic recombination, and their impacts on adaptation over evolutionary time scales, are exerted largely through hotspot-regulating DNA sites and their binding/activator proteins.

## Conclusion

From our perspective, the punctate distribution of meiotic recombination across genomes and the striking plasticity of recombination landscapes are each readily explained by the fact that many, distinct DNA sites each position recombination at hotspots. Each class of hotspots functions as an independently and precisely controlled rheostat; together, they can dramatically remodel the recombination landscape in response to changing conditions.

There are many interesting questions for future study. For example, are the fundamental mechanisms of plasticity discovered in fission yeast conserved throughout eukaryotes? To what extent do hotspot DNA site-mediated, evolutionarily instantaneous changes in the recombination landscape contribute to the observed differences between related species, subspecies and isolated populations? Similarly, what fraction of the differences in recombination landscapes become fixed during isolation or speciation (e.g., by drift or divergence in DNA sites, binding/activator proteins, and regulatory signal transduction networks)? Lastly, are the molecular mechanisms for rapid remodeling (adaptation) in the frequency distribution of recombination also adaptive (beneficial) over evolutionary time scales?

## Data Availability

No new data were generated for this perspective article. Published data were replotted in Figure 1D (Zhang et al., 2017) and Figure 2C (Protacio et al., 2022). Figure 1B and Figure 2B were reproduced with permission under CC BY-NC 4.0 from (Fowler et al., 2014); Figure 1C with permission under CC BY 4.0 from (Shanfelter et al., 2019); Figures 2D,E with permission under CC BY from (Protacio et al., 2022). Images were reproduced with minor modifications (cropping, scaling and labeling) for clarity and parallel construction.
